# Supplementary material for: Investigating the work–life experiences of nursing faculty in Canadian academic settings and the factors that influence their retention: protocol for a mixed-method study
Source: BMJ Open. 2022 Jan 3;12(1):e056655. doi: 10.1136/bmjopen-2021-056655 (PMC8724810; doi:10.1136/bmjopen-2021-056655)
Supplement: Author's manuscript [file bmjopen-2021-056655.draft_revisions.pdf]

BMJ Open

BMJ Open is committed to open peer review. As part of this commitment we make the peer review history of every article we publish publicly available.

When an article is published we post the peer reviewers' comments and the authors' responses online. We also post the versions of the paper that were used during peer review. These are the versions that the peer review comments apply to.

The versions of the paper that follow are the versions that were submitted during the peer review process. They are not the versions of record or the final published versions. They should not be cited or distributed as the published version of this manuscript.

BMJ Open is an open access journal and the full, final, typeset and author-corrected version of record of the manuscript is available on our site with no access controls, subscription charges or pay-per-view fees (http://bmjopen.bmj.com).

If you have any questions on BMJ Open's open peer review process please email info.bmjopen@bmj.com

Worklife study protocol: A cross-sectional study exploring perspectives of nursing faculty in Canada

Journal:	*BMJ Open*
Manuscript ID	bmjopen-2021-056655
Article Type:	Protocol
Date Submitted by the Author:	23-Aug-2021
Complete List of Authors:	Boamah, Sheila A.; McMaster University Faculty of Health Sciences, Nursing
Keywords:	Human resource management < HEALTH SERVICES ADMINISTRATION & MANAGEMENT, Organisational development < HEALTH SERVICES ADMINISTRATION & MANAGEMENT, Quality in health care < HEALTH SERVICES ADMINISTRATION & MANAGEMENT

SCHOLARONE™
Manuscripts

Title page

Worklife study protocol: A cross-sectional study exploring perspectives of nursing faculty in Canada

Author details
Sheila A. Boamah [1,*]

Author Affiliations
[1]School of Nursing, Faculty of Health Sciences, Master University, 1280 Main Street West, Hamilton, ON, L8S 4K1, Canada.

*** Correspondence**
Dr. Sheila A. Boamah; boamahs@mcmaster.ca

For peer review only

Declarations

Twitter: Sheila A. Boamah. *@SABoamah*

Acknowledgements: The author wishes to thank Drs. Arku and Djiadeu for reviewing the manuscript draft.

Funding: This work was supported by the Social Science and Humanities Research Council (SSHRC) of Canada, grant number [430-2020-01042].

Competing interests: None declared.

Patient consent for publication: Not required.

Patient consent for publication: Patients or the public were not involved in the design, or conduct, or reporting, or dissemination plans of our research.

Provenance and peer review: Not commissioned; externally peer reviewed.

Open access: This is an open access article distributed in accordance with the Creative Commons Attribution Non-Commercial (CC BY-NC 4.0) license, which permits others to distribute, remix, adapt, build upon this work non-commercially, and license their derivative works on different terms, provided the original work is properly cited, appropriate credit is given, any changes made indicated, and the use is non-commercial. See: http://creativecommons.org/licenses/by-nc/4.0/.

ORCID iD
Sheila A. Boamah https://orcid.org/0000-0001-6459-4416

ABSTRACT

Introduction: While all research-oriented faculty face the pressures of academia, female faculty in fields including science, engineering, medicine, and nursing, are especially susceptible to burnout. Nursing is unique in that, it remains a predominantly female-dominated profession which implies that there is a critical mass of females who are disproportionately affected by this imbalance. To date, little is known about the experiences of nursing faculty especially, new and early career researchers and the factors that influence their retention. This study aims to understand the worklife experiences of nursing faculty in Canadian academic settings.

Methods and Analysis: A mixed-methods design will be used in this study. For the quantitative data, we will survey a sample of new and early career nursing faculty across Canadian academic institutions. Eligible participants will be invited to complete the Web-based structured questionnaire in both French and English language. Data will be evaluated using generalized linear regression model and structural equation modeling. Given the complexities of worklife issues in Canada, qualitative focus group interviews will also be conducted. Emerging themes will be integrated with the survey findings and used to enrich the interpretation of the quantitative data.

Ethics and Dissemination: This study has received ethical approval from the Hamilton Integrated Research Ethics Board. The study findings will be disseminated to academics and non-academic stakeholders through national and international conference presentations and peer-reviewed open-access journals. A user-friendly report will be shared with professional nursing associations such as the Canadian Associations of Schools of Nursing, and through public electronic forums (e.g., Twitter). Evidence from this study will also be shared with stakeholders including senior academic leaders and health practitioners, government, and health service policy makers, to raise the profile of discourses on the nursing workforce shortages; and women's worklife balance, a public policy issue often overlooked at the national level. Such discussion is especially pertinent in light of the disproportionate impact of COVID-19 on women, and female academics. The findings will be used to inform policy options for improving nursing faculty retention in Canada and globally.

For peer review only

Strengths and limitations of this study

- This will be the first national study examining the worklife experiences of nursing faculty in Canada.
- By adopting a two-phase sequential explanatory mixed-methods design, we will explore in-depth the dichotomy of work and non-work life and strategies to improve faculty retention.
- While the present study focuses on nursing faculty wellness and retention and is situated in Canada, the findings can inform policies and practices in other academic disciplines and countries facing similar challenges.
- More broadly, our study will raise the profile of discourse on women's worklife balance in Canada and beyond.

For peer review only

BACKGROUND

The debate over the causes of the underrepresentation of women in academia seems to divide the potential causal factors into gender-based versus structural inequity. The presence and status of women faculty is 'a pressing national issue for reasons of social equity (or inequity) in access to, and rewards gained in', the workforce.[1] While all research-oriented faculty face the pressures of academia, female faculty are especially susceptible to burnout.[2, 3, 4] Female faculty in fields including science, engineering, medicine, and nursing often struggle with balancing career and family, and gaining the respect of students and colleagues.[1, 5, 6] Nursing is unique in that, it remains a predominantly female-dominated profession which implies that there is a critical mass of females who are disproportionately affected by this imbalance.[7, 8] Additionally, most nurse academics come from a clinical background with little preparation for the complex faculty role and understanding of the distinct academic culture, language, expectations, values, and behaviours.[9, 10] Given the extensive history of nursing practice and education in Canada, we still do not have a commensurate understanding of faculty retention.

Currently, nursing in Canada is plagued by recurrent faculty shortage. This shortage directly impacts the supply and demand of registered nurses (RNs) in the clinical work environments and the ability of nurses to deliver high quality patient care. In 2016, only 19.3% of faculty members employed by schools of nursing in Canada held a permanent position.[11] This shortage is fueled, in part, by a rapidly aging workforce, an undersupply of doctorally prepared nurses to take their place, lack of qualified applicants, and poor work environment.[12–15] About 40% of Canadian nursing faculty are over the age of 55 and 17.8% are eligible to retire.[11] The retirement projections of the aging faculty limit the pool of doctorally prepared faculty as fewer nurses are applying for graduate programs.[15–18] According to Canadian Associations of Schools of Nursing,[11] the number of graduate students currently enrolled in nursing schools is far less than what is required to meet the demands for advance practice in clinical settings and in academia, and this is contributing to exponential growth in unoccupied faculty positions across Canada. This is exacerbated by the fact that only 60% of nurses with doctorates, choose the academic role.[19] Across Canada, schools of nursing identified shortage of qualified applicants, non-competitive salaries, and lack of funds to create permanent positions as the three main factors limiting their ability to recruit new faculty.[19, 20] This results in heavy workloads for employed faculty, their inability to train sufficient graduate students, lack of quality time for students in the program, contributing to longer graduation periods, faculty burnout, worklife conflict, dissatisfaction, and further attrition.[17, 21, 22]

With the concurrent nursing faculty shortage and the impact of the COVID-19 pandemic on the nursing workforce, attracting and retaining quality faculty is extremely important to educational institutions as low faculty retention rate might create both monetary and academic consequences.[23] The predominant discourse on nursing faculty recruitment and retention centers on the realities of an aging workforce, lucrative opportunities in the clinical and private sectors, heavy workloads, poor work environment, lack of organizational support,[24–26] and unrealistic role expectations.[22, 27, 28] What is missing in this discourse is a nuanced understanding of the joint effect of multiple worklife issues on recruitment and retention of early career researchers (ECRs)/academics, and comprehensive strategies to alleviate the shortage of faculty. Additionally, past studies on nursing faculty have mainly been in the US context with little focus in Canada.[25, 29] There is limited understanding, at all administrative levels, regarding the impact of institutional worklife issues including heavy workloads, long working hours, and lack of

For peer review only - http://bmjopen.bmj.com/site/about/guidelines.xhtml

mentorship on nursing faculty members' satisfaction, and subsequent implications on their intentions to leave their job.[10]

Using Structuration theory[30] to explore the interplay between individuals and organizations, we seek to understand worklife experiences of nurse academics, especially ECRs and the factors they identify as relevant to their success. Exploring the perspectives, concerns, and experiences of nursing faculty, especially ECRs, is important in academia in terms of succession planning. Thus, the overall **goal of this study** is to investigate nursing faculty's worklife experiences in Canadian academic settings and the factors that influence their retention.

Objectives

The primary objectives of this study are to: (1) assess the facilitators and barriers that influence new and early career faculty's successful transition to the academic role; (2) examine the personal and situational factors that jointly influence nursing faculty's satisfaction and intentions to remain in their jobs; (3) delineate the comparative experiences of female and male academics; (4) examine how institutional leadership impacts new faculty's quality of worklife, and productivity; and (5) explore nursing faculty's understanding of worklife integration and balance.

Theoretical perspectives

Guided by the overall goal, the proposed study is grounded in the *Structuration theory*,[30] and the relational interdependence of structure and agency (see Figure 1). The use of structuration as the theoretical lens in this research offers a multidimensional approach that takes into account the various underlying components of the "structure" which is a set of rules and resources that enable and constrain decisions, choice, action, and thought, and "agency" referring to the ability of an individual to take action, with an appreciation for their interrelatedness.[31] Structuration theory takes a dualistic view of social worlds by considering both the members of the organization (actors) and the organization's structure, and the reality of the tensions within this dialectic and the dynamic and transformational processes inherent within organizations.[30] The actions that individuals in any organization take to meet their work and non-work demands (e.g., childcare) at a particular time are shaped by the structural properties of that context (e.g., resources, institutions).

Structuration theory is a powerful framework for studying the qualities of human relationships and a productive theoretical lens to apply to this study of worklife issues in academia particularly because it allows us to consider the different levels of influence that affect new faculty's retention. Individuals' actions and interactions create or recreate the lower-level structures (e.g., at the family level), and therefore reinforce the higher-level structures at the level of organizations.[32] In other words, individuals tend to create the kinds of family structures that fit the organizational expectations as they are created. Several researchers[32–34] have measured "how" structures (e.g., leadership) can influence employees' ability to be satisfied and productive at work. The literature indicates that leadership influences the alignment of individual behaviour in an organization. Effective leadership has been shown to contribute to high morale in workplace which results in increased satisfaction and organizational commitment.[35–38] However, it is unclear how institutional leaders may bring about behavioural changes in academia. This study seeks to address this gap and the evidence from this research will add to the theoretical basis for extending Giddens's structuration theory to incorporate leadership as a precursor of faculty satisfaction and retention.

For peer review only

The proposed study seeks to adapt and strengthen the use of this theory by exploring the role and effects of both structural/workplace and personal factors on individual's adjustment to the work environment, and the multiple dimensions of organizational and career satisfaction. By situating this study on faculty worklife within the structuration framework, evidence from this research on how the interplay of personal and situational factors culminate in either retention or attrition will have broader implications pertaining to the worklife debate among women in the Canadian workforce and beyond.

<Insert Figure 1 here>

For peer review only

METHODOLOGY

Design

We will adopt a two-phase sequential explanatory mixed-methods design — a cross-sectional survey followed by qualitative focus group interviews to answer the research objectives, given their combined strength in addressing the complexities of worklife issues in Canada.[39] The phases of the study including recruitment and data collection procedures are outlined in Figure 2.

Phase 1: Quantitative methods and analysis

The first phase of the study will focus on the quantitative survey, which will help to address ***Objectives 1, 2, 3,*** and ***4***. A comprehensive literature review on quality of work environment was conducted prior to the study which will be used to inform the survey design.

Sample

Participants will comprise of nursing faculty working at Canadian colleges and universities, including ECRs (tenure-track faculty) within their first five-seven years in academia. The 5-7 timeframe was chosen based on the tenure review process adopted by most Canadian universities.[40] Based on the estimated number of new and ECRs/academics in Canada,[19] we anticipate a sample of 200 faculty members who may be eligible to participate in this study – a sufficient sample size to obtain reliable parameter estimates among variables in the proposed conceptual framework for this study.[41]

Recruitment and data collection

An email invitation will be sent to eligible faculty members using their publicly available e-addresses to complete a Web-based structured questionnaire offered in both Canadian national languages – English and French. The tailored design method (TDM) for Internet surveys[42] will be used to promote participation of faculty to ensure an adequate sample size and representative sample. The TDM involves a set of procedures intended to create respondent trust and perceptions of increased rewards with the overall goal of reducing survey error. The questionnaire will be hosted securely online using our institutional site licensed Qualtrics survey software and will not include personal identifiers to assure anonymity and confidentiality.

Measures

Among other broad topics, the questionnaire will cover workload issues, mentorship, leadership practices, organizational support, and job satisfaction. In total, 7 standardized instruments will be used, including the well-validated Multifactor Leadership Questionnaire,[43, 44] Job Satisfaction Survey,[45] and Work Interference with Personal Life (WIPL).[46–48] There will be 30 questions in total. Sample items from the WIPL include: 'My job makes it difficult to maintain the kind of personal life I would like' and 'I often neglect my personal needs because of the demands of my work.' Issues of reliability and validity as well as survey length to minimize respondent burden will be considered in selecting the final survey items. Demographic data including age, gender, ethnicity, highest education, academic rank, and tenure status will be collected.

Data analysis and interpretation

Descriptive statistics will be used to profile the characteristics of new nursing faculty, and to analyze bivariate relationships using SPSS (v.26) software.[49] Rigorous methods will be used to verify the accuracy and quality of the measurement tools by performing confirmatory factor analysis and Cronbach's alpha reliabilities. Given the complexity of the research questions, our target is to employ structural equation modelling (SEM) technique with maximum likelihood estimation to test the fit between the data and the hypothesized model in Mplus software (v.7.3).[41, 50] SEM is a powerful multivariate technique which allows us to examine multiple relationships among variables in a single model.[51]

<Insert Figure 2 here>

Phase 2: Qualitative methods and analysis

A major critique of quantitative surveys, including the Web-based self-report survey, on worklife issues is that they do not provide in-depth accounts of the work experiences of pre-tenured faculty.[39] Therefore, in second phase of this study, we will undertake qualitative descriptive research (focus group interviews), to complement and enrich the quantitative findings by probing in greater detail the circumstances leading to potential turnover. This component of the study will address ***Objective 5***.

Recruitment and data collection

Focus group discussions have been characterized as a microcosm of "the thinking society", capable of revealing the processes whereby social norms are collectively shaped through debate and argument.[52, 53] As such, the proposed study will make use of focus group discussion. Our focus group will provide a forum for ECRs and other academics to critically discuss a range of issues including worklife balance, workload demands, supports, and resources. We will conduct semi-structured focus group interview of about 60 minutes in duration with approximately 20-25 survey respondents who volunteers to participate in the qualitative phase of the study. There would be five focus groups, consisting of a mix of all gender including male and female nurse academics. Each focus group discussion will have 5 members (n=25). The number of focus group attendees in this study is informed by the requirements of thematic saturation,

For peer review only

which is the point at which themes become repetitive.[54] Faculty members will be interviewed in-person as face-to-face interviews offer the advantages of rapport and visual cues,[55] and those outside the local geographical region will be interviewed virtually via Zoom or any other communication medium of their choice.

Focus group interview guide

An interview guide will be developed stemming from the Phase 1 of the study. Sample focus group questions include: 'Tell us about your first few months/years as a new professor' and 'What do you believe has had the most impact on your transition to the academic role?' After obtaining informed consent from participants, the focus group discussion will be audio recorded using Audacity® and transcribed verbatim, and the research team will perform a textual analysis of emerging themes and conceptual categories, while also looking for patterns and relationships.

Data analysis and interpretation

Initially, data analysis will focus on understanding the information, then identifying codes and categories. Thematic analysis will be conducted using open-coding techniques.[56] Codes will be applied to statements that share commonalities. Topics of discussion in response to questions, areas of agreement and disagreement, and characteristics of the discussion will be examined.[57] In addition to themes that emerges from the data, other themes (e.g., demands of teaching, and lack of clarity about tenure requirements) not included in the preliminary framework will be included in analysis inductively and the framework will be modified accordingly. The qualitative data analysis software, N-Vivo (v.12),[58] will be used extensively for data management and coding of text. Due attention will be given to rigour in this study. To maintain a high level of quality, we will utilize methodological (interviews and survey) and theory triangulation, keep audit trails, and engage in bi-weekly debriefing sessions. Data from the observations and interviews will be anonymized, stored securely at the institutional research data repository throughout the data life (5 years).

Patient and public involvement

Patients and/or the public were not involved in the design, or conduct, or reporting, or dissemination plans of this research. The dissemination of the study results will be discussed in subsequent section.

DISCUSSION

Although there are emerging studies on nursing faculty worklife,[2, 18, 25] the perspectives and experiences of new and ECRs are largely missing from the current debates on the issue. This perspective is vital because early career faculty are the next generation of leaders in academia, and they can provide valuable new insights into the successes and challenges of retention in the long term. Considering the views of new faculty brings to the fore the necessity of thinking in terms of succession planning. Although the nursing faculty shortage is a multifaceted problem, a few existing studies of the crises are descriptive in nature and most studies are limited to single sites.

Our goal is to understand the issue from a holistic perspective, which make this study innovative in a number of levels: First, it aims to combine quantitative and qualitative

For peer review only

methodologies in novel ways to gain a nuanced understanding of faculty members' worklife experiences and how academic leadership influences new faculty recruitment and retention. Second, the focus on academic leadership is unique in that less is known about how leadership of nursing faculties influence new nurse academics' quality of worklife, satisfaction, and subsequent retention.[18] Third, this research aims to examine the dynamic interplay of leadership, work environment, and satisfaction by jointly focusing on: (i) individual-level characteristics including personal and psychological traits; (ii) structural characteristics including institutional support and culture, and (iii) Psychosocial attributes (e.g., coping, support) to provide sophisticated and comprehensive view of the transitional experiences of new faculty and resources that will aid in their success. The existing research on nursing faculty experiences is in the US context and very little on the Canadian context. Furthermore, there is paucity of research on the ECRs' experiences and how that culminates in retention.

The proposed study will make substantive contributions to both research and practice on nursing faculty recruitment and retention, which can serve as a baseline to address the national and global nursing faculty shortage. The findings will expand our knowledge on how leadership impact retention decision making in the academic context, as well as in the organizational literature. The outcomes of this study will be valuable for nursing faculty planning and decision making in the medium to long term; and will advance worklife research by using evidence-based planning to explore informed policymaking, at a broader, conceptual level and in the specific case of recruiting and retention as a proxy for local and national policies. Furthermore, outcomes will benefit both policymakers and health practitioners by contributing to our knowledge of the policy development, implementation, and evaluation process.

While this study focuses on faculty recruitment and retention and is situated in Canada, the goal is to develop an informed policymaking framework that is applicable in the international contexts, especially countries facing similar challenges. More fundamentally, our research will raise the profile of discourses on women's worklife balance, a public policy issue often overlooked at the national level. Such discussion is especially pertinent in light of the disproportionate impact of COVID-19 on women, and female academics. Specifically, our study will highlight the critical role of informed/evidence-based planning plays in society, and its importance for stabilizing and enhancing recruitment and retention of new faculty. The findings will make an original contribution to worklife studies by shedding light on important challenges, considerations, and strategies for addressing the current nursing faculty shortage in Canada. Overall, the proposed study will contribute to a gap in the literature and enhance the current evidence on nursing education and faculty wellbeing in Canada.

ETHICS AND DISSEMINATION

This study has been approved by the Hamilton Integrated Research Ethics Board (HiREB) (#1477). To ensure the privacy, dignity, integrity, and anonymity of the participants, only group data will be reported.

To ensure the findings have wide accessibility and impact, our knowledge mobilization (KM) plan involves diverse strategies (e.g., stakeholder engagements, publications, conferences). Target audiences for this project are policymakers and influencers (e.g., government officials, practitioners, academics, health and labour unions). The purpose of our KM plan is to both provide these audiences with timely information about our project and create meaningful exchange of ideas among these groups. KM will be ongoing throughout the project. Our

For peer review only

accessibility plan is focused on presenting our findings in different media. The study outputs will be presented at academic and non-academic conferences, through open access peer-review publications, and media release to reach the widest audiences, including interested laypeople and the general public. Academic and international audiences will be reached mainly through journal publications and conference presentations and a user-friendly report will be shared with nursing associations and health agencies. Further, we will make use of social media (e.g., Twitter) for connecting and networking these audiences.

For peer review only

References

1. Fox MF. Women and men faculty in academic science and engineering: Social-organizational indicators and implications. American Behavioral Scientist. 2010;53(7).
2. Candela L, Gutierrez A, Keating S. A national survey examining the professional work life of today's nursing faculty. Nurse Education Today. 2013;33(8).
3. Reybold LE, Alamia JJ. Academic transitions in education: A developmental perspective of women faculty experiences. Journal of Career Development. 2008;35(2).
4. Ward K, Wolf-Wendel L. Academic Motherhood: Managing Complex Roles in Research Universities. Vol. 27, Review of Higher Education. 2004.
5. Howe-Walsh L, Turnbull S. Barriers to women leaders in academia: tales from science and technology. Studies in Higher Education. 2016;41(3).
6. Xu YJ. Gender disparity in STEM disciplines: A study of faculty attrition and turnover intentions. Research in Higher Education. 2008;49(7).
7. Canadian Institute for Health Information [CIHI]. Nursing in Canada, 2018: A Lens on Supply and Workforce. Ottawa, ON: CIHI; 2019.
8. Simpson R. Men in non-traditional occupations: Career entry, career orientation and experience of role strain. Gender, Work and Organization. 2005;12(4).
9. Cleary M, Horsfall J, Jackson D. Mental Health Nursing: Transitions from practice roles to academia. Vol. 47, Perspectives in Psychiatric Care. 2011.
10. Reid TP, Hinderer KA, Jarosinski JM, Mister BJ, Seldomridge LA. Expert clinician to clinical teacher: Developing a faculty academy and mentoring initiative. Nurse Education in Practice. 2013;13(4).
11. Canadian Association of Schools of Nursing [CASN]. Registered Nurses Education in Canada Statistics. Ottawa, ON.; 2016.
12. American Association of Colleges of Nursing. [AACN]. Nurse faculty shortage. 2014.
13. Canadian Association of Schools of Nursing. [CASN]. Registered Nurses Education in Canada Statistics 2009-2010. 2012.
14. Kaufman K. Introducing the NLN/Carnegie National Survey of Nurse Educators: compensation, workload, and teaching practice. Nursing education perspectives. 2007;28(3).
15. McDermid F, Peters K, Jackson D, Daly J. Factors contributing to the shortage of nurse faculty: A review of the literature. Nurse Education Today. 2012;32(5).
16. Siela D, Twibell KR, Keller V. The shortage of nurses and nursing faculty: What critical care nurses can do. AACN Advanced Critical Care. 2008;19(1).
17. Tourangeau AE, Wong M, Saari M, Patterson E. Generation-specific incentives, and disincentives for nurse faculty to remain employed. Journal of Advanced Nursing. 2015;71(5).
18. Vandyk A, Chartrand J, Beké É, Burlock L, Baker C. Perspectives from Academic Leaders of the Nursing Faculty Shortage in Canada. International Journal of Nursing Education Scholarship. 2017;14(1).
19. Canadian Association of Schools of Nursing [CASN]. Registered Nurses Education in Canada Statistics: 2012-2013. Ottawa; 2014.
20. Tourangeau A, Saari M, Patterson E, Ferron EM, Thomson H, Widger K, et al. Work, work environments and other factors influencing nurse faculty intention to remain employed: A cross-sectional study. Nurse Education Today. 2014;34(6).

For peer review only

21. Gerolamo AM, Roemer GF. Workload and the nurse faculty shortage: Implications for policy and research. Nursing Outlook. 2011;59(5).
22. Yedidia MJ, Chou J, Brownlee S, Flynn L, Tanner CA. Association of faculty perceptions of work–life with emotional exhaustion and intent to leave academic nursing: Report on a national survey of nurse faculty. Journal of Nursing Education. 2015;53(10).
23. Rensselaer Polytechnic Institute. Faculty retention proves a major challenge for universities. 2012.
24. Beckmann CA, Cannella BL, Wantland D. Faculty Perception of Bullying in Schools of Nursing. Journal of Professional Nursing. 2013;29(5).
25. Boamah SA, Callen M, Cruz E. Nursing faculty shortage in Canada: A scoping review of contributing factors. Nursing Outlook. 2021.
26. Smeltzer SC, Sharts-Hopko NC, Cantrell MA, Heverly MA, Wise NJ, Jenkinson A, et al. Challenges to research productivity of doctoral program nursing faculty. Nursing Outlook. 2014;62(4).
27. Gormley DK, Kennerly S. Influence of Work Role and Perceptions of Climate on Faculty Organizational Commitment. Journal of Professional Nursing. 2010;26(2).
28. McDonald PJ. Transitioning from clinical practice to nursing faculty: Lessons learned. Vol. 49, Journal of Nursing Education. 2010.
29. Gerolamo AM, Overcash A, McGovern J, Roemer G, Bakewell-Sachs S. Who will educate our nurses? A strategy to address the nurse faculty shortage in New Jersey. Nursing Outlook. 2014;62(4).
30. Giddens A. The constitution of society: Outline of the theory of structuration: Elements of the theory of structuration. In: Practicing History: New Directions in Historical Writing after the Linguistic Turn. 2004.
31. Orlikowski WJ. The Duality of Technology: Rethinking the Concept of Technology in Organizations. Organization Science. 1992;3(3).
32. Trefalt Š, Drnovšek M, Svetina-Nabergoj A, Adlešič RV. Work-life experiences in rapidly changing national contexts: Structural misalignment, comparisons, and choice overload as explanatory mechanisms. European Management Journal. 2013;31(5).
33. Festing M, Maletzky M. Cross-cultural leadership adjustment - A multilevel framework based on the theory of structuration. Human Resource Management Review. 2011;21(3).
34. Place KR, Vardeman-Winter J. Where are the women? An examination of research on women and leadership in public relations. Public Relations Review. 2018;44(1).
35. Bass BM. Transformational leadership: Industrial, military, and educational impact. Transformational Leadership Industry Military and Educational Impact. 1998.
36. Boamah SA. The influence of transformational leadership on nurse-reported patient safety outcomes. Electronic Thesis and Dissertation Repository 4401 Western University, London, ON http://ir.lib.uwo.ca/etd/4401. 2017.
37. Cummings GG, MacGregor T, Davey M, Lee H, Wong CA, Lo E, et al. Leadership styles and outcome patterns for the nursing workforce and work environment: a systematic review. International journal of nursing studies. 2010;47(3):363–85.
38. Giddens J. Transformational leadership: What every nursing dean should know. Journal of Professional Nursing. 2018;34(2).
39. Sandelowski M. Whatever happened to qualitative description? Research in Nursing & Health. 2000;23(4).

For peer review only

40. Gravestock P, Greenleaf EG. Overview of tenure and promotion policies across Canada. 2018.
41. Kline RB. Principles and practice of structural equation modeling. New York, NY 10012: The Guilford Press; 2011.
42. Dillman DA. Mail and Internet surveys: The tailored design method--2007 Update with new Internet, visual, and mixed-mode guide. John Wiley & Sons; 2011.
43. Avolio BJ, Bass BM. Multifactor Leadership Questionnaire manual. Redwood City. CA: Mind Garden, editor. 2014.
44. Boamah SA, Tremblay P. Examining the Factor Structure of the MLQ Transactional and Transformational Leadership Dimensions in Nursing Context. Western Journal of Nursing Research. 2019;41(5).
45. Hackman, J. R., and Oldham GR. The Job Diagnostic Survey: An Instrument for the Diagnosis of Jobs and the Evaluation of Job Redesign Projects. Report No 4, Yale University, Department of Administration Science, New Haven, CT. 1974;(1).
46. Boamah S, Laschinger H. Engaging new nurses: the role of psychological capital and workplace empowerment. Journal of Research in Nursing. 2014;1744987114527302.
47. Boamah SA, Laschinger H. The influence of areas of worklife fit and work-life interference on burnout and turnover intentions among new graduate nurses. Journal of Nursing Management. 2016;24(2).
48. Greenhaus JH, Allen TD, Spector PE. Health Consequences of Work-Family Conflict: The Dark Side of the Work-Family Interface. Vol. 5, Research in Occupational Stress and Well Being. 2006.
49. SPSS Incorporated. SPSS for Windows Standard Version 26.0. IBM SPSS Inc.; 2021.
50. Muthén L, Muthén B. Mplus user's guide (6th ed.). Los Angeles. 2012.
51. Mackinnon DP, Lockwood CM, Williams J. Confidence Limits for the Indirect Effect: Distribution of the Product and Resampling Methods. Multivariate behavioral research. 2004;39(1):99–128.
52. Lunt P, Livingstone S. Rethinking the Focus Group in Media, and Communications Research. Journal of Communication. 1996;46(2).
53. Morgan DL. Focus Group. New York, NY: Oxford University Press; 2004.
54. Strauss A, Corbin J. Basics of Qualitative Research: Techniques and Grounded Theory Procedures for Developing Grounded Theory. The Modern Language Journal. 1998.
55. Sullivan JR. Skype: An Appropriate Method of Data Collection for Qualitative Interviews? The Hilltop Review. 2012;6(1).
56. Crang M. Qualitative methods: There is nothing outside the text? Progress in Human Geography. 2005;29(2).
57. Lavis JN, Robertson D, Woodside JM, McLeod CB, Abelson J. How can research organizations more effectively transfer research knowledge to decision makers? Milbank Quarterly. 2003;81(2).
58. Edhlund BM. NVivo 10 essentials: your guide to the world's most powerful data analysis software. Stallarholmen, Sweden: Form & Kunskap AB. 2012.

For peer review only

Figure 1

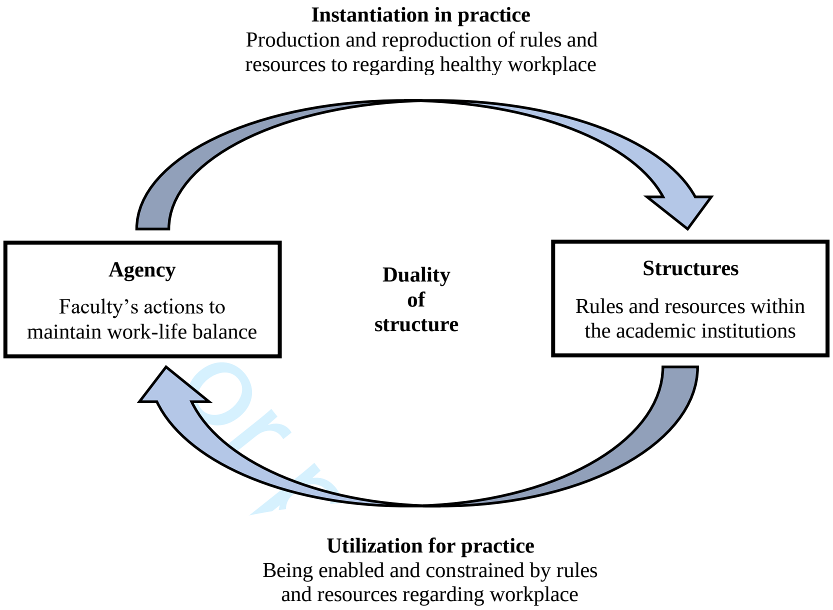

Fig. 1. Structuration model of the intersection of nursing academy and worklife balance (Adapted from Giddens, 1984)

For peer review only

Figure 2

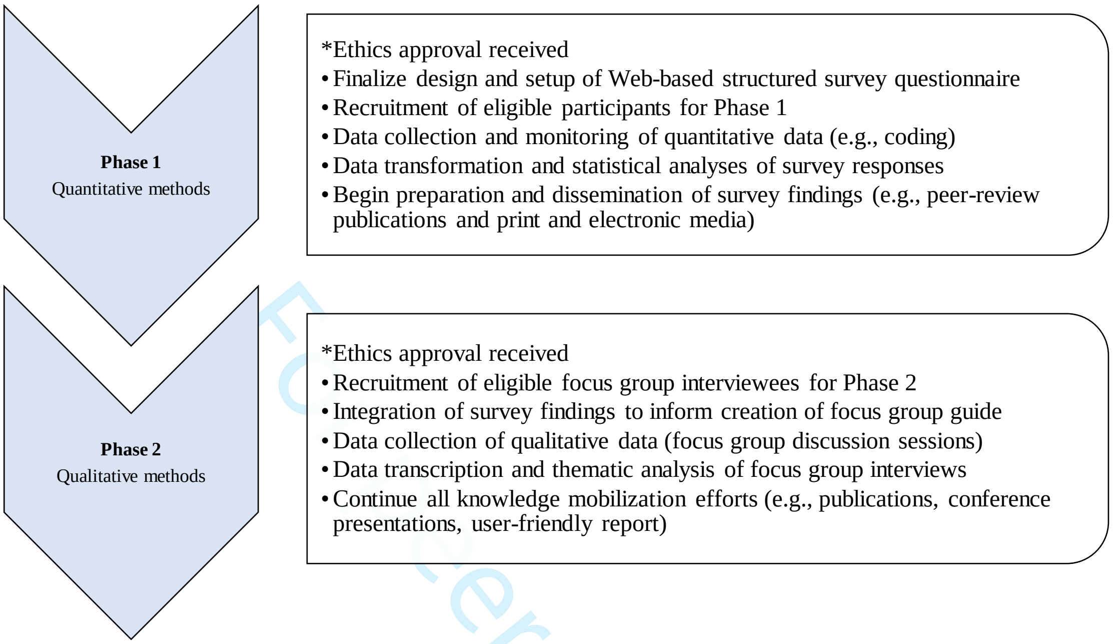

Fig. 2. Phases of study protocol

For peer review only

BMJ Open

Investigating the work-life experiences of nursing faculty in Canadian academic settings and the factors that influence their retention: protocol for a mixed- methods study

Journal:	*BMJ Open*
Manuscript ID	bmjopen-2021-056655.R1
Article Type:	Protocol
Date Submitted by the Author:	16-Nov-2021
Complete List of Authors:	Boamah, Sheila A.; McMaster University Faculty of Health Sciences, Nursing
**Primary Subject Heading**:	Health services research
Secondary Subject Heading:	Nursing
Keywords:	Human resource management < HEALTH SERVICES ADMINISTRATION & MANAGEMENT, Organisation of health services < HEALTH SERVICES ADMINISTRATION & MANAGEMENT, Quality in health care < HEALTH SERVICES ADMINISTRATION & MANAGEMENT

SCHOLARONE™
Manuscripts

Title page

Investigating the work-life experiences of nursing faculty in Canadian academic settings and the factors that influence their retention: protocol for a mixed-methods study

Author details
Sheila A. Boamah [1,*]

Author Affiliations
[1]School of Nursing, Faculty of Health Sciences, Master University, 1280 Main Street West, Hamilton, ON, L8S 4K1, Canada.

*** Correspondence**
Dr. Sheila A. Boamah; boamahs@mcmaster.ca

For peer review only

Declarations

Twitter: Sheila A. Boamah. *@SABoamah*

Acknowledgements: The author wishes to thank Drs. Arku and Djiadeu for reviewing the initial manuscript draft.

Funding: This work was supported by the Social Science and Humanities Research Council (SSHRC) of Canada, grant number [430-2020-01042].

Competing interests: None declared.

Patient consent for publication: Not required.

Provenance and peer review: Not commissioned; externally peer reviewed.

Open access: This is an open access article distributed in accordance with the Creative Commons Attribution Non-Commercial (CC BY-NC 4.0) license, which permits others to distribute, remix, adapt, build upon this work non-commercially, and license their derivative works on different terms, provided the original work is properly cited, appropriate credit is given, any changes made indicated, and the use is non-commercial. See: http://creativecommons.org/licenses/by-nc/4.0/.

ORCID iD
Sheila A. Boamah https://orcid.org/0000-0001-6459-4416

ABSTRACT

Introduction: While all research-oriented faculty face the pressures of academia, female faculty in fields including science, engineering, medicine, and nursing, are especially susceptible to burnout. Nursing is unique in that, it remains a predominantly female-dominated profession, which implies that there is a critical mass of females who are disproportionately affected and/or at higher risk of burnout. To date, little is known about the experiences of nursing faculty especially, new and early career researchers and the factors that influence their retention. This study aims to understand the work-life (the intersection of work with personal life) experiences of nursing faculty in Canadian academic settings and the factors that influence their retention.

Methods and Analysis: A mixed-methods design will be used in this study. For the quantitative study, a sample of approximately 1500 new and early career nursing faculty across Canadian academic institutions will be surveyed. Eligible participants will be invited to complete a Web-based structured questionnaire in both French and English language. Data will be evaluated using generalized linear regression model and structural equation modeling. Given the complexities of work-life issues in Canada, qualitative focus group interviews with about 20-25 participants will also be conducted. Emerging themes will be integrated with the survey findings and used to enrich the interpretation of the quantitative data.

Ethics and Dissemination: This study has received ethical approval from the Hamilton Integrated Research Ethics Board (#1477). Prior to obtaining informed consent, participants will be provided with information about study risks and benefits and strategies undertaken to ensure confidentiality and anonymity. The study findings will be disseminated to academics and non-academic stakeholders through national and international conference presentations and peer-reviewed open-access journals. A user-friendly report will be shared with professional nursing associations such as the Canadian Associations of Schools of Nursing, and through public electronic forums (e.g., Twitter). Evidence from this study will also be shared with stakeholders including senior academic leaders and health practitioners, government, and health service policy makers, to raise the profile of discourses on the nursing workforce shortages; and women's work-life balance, a public policy issue often overlooked at the national level. Such discussion is especially pertinent in light of the disproportionate impact of COVID-19 on women, and female academics. The findings will be used to inform policy options for improving nursing faculty retention in Canada and globally.

For peer review only

Strengths and limitations of this study

- This will be the first national study examining the work-life experiences of nursing faculty in Canada.
- By adopting a two-phase sequential explanatory mixed-methods design, the proposed study will explore in-depth the dichotomy of work and non-work life and strategies to improve faculty retention; and more broadly, raise the profile on discourse on women's work-life balance in Canada and beyond.
- While the present study focuses on nursing faculty wellness and retention and is situated in Canada, the findings can inform policies and practices in other academic disciplines and countries facing similar challenges.
- The cross-sectional design will not conclusively support causation to the evidence of co-variation in the study variables.
- The subjective nature of qualitative research has potential for recall bias thus, critical attention should be paid to rigor and trustworthiness.

For peer review only

INTRODUCTION

The debate over the causes of the underrepresentation of women in academia seems to divide the potential causal factors into gender-based versus structural inequity. The presence and status of women faculty is 'a pressing national issue for reasons of social equity (or inequity) in access to, and rewards gained in', the workforce.[1] While all research-oriented faculty face the pressures of academia, female faculty are especially susceptible to burnout.[2–4] Female faculty in fields including science, engineering, medicine, and nursing often struggle with balancing career and family, and gaining the respect of students and colleagues.[1,5,6] Nursing is unique in that, it remains a predominantly female-dominated profession which implies that there is a critical mass of females who are disproportionately affected by this imbalance.[7,8] Additionally, most nurse academics come from a clinical background with little preparation for the complex faculty role and understanding of the distinct academic culture, language, expectations, values, and behaviours.[9,10] Given the extensive history of nursing practice and education in Canada, there is still not a commensurate understanding of faculty retention.

Currently, nursing in Canada is plagued by recurrent faculty shortage. This shortage directly impacts the supply and demand of registered nurses (RNs) in the clinical work environments and the ability of nurses to deliver high quality patient care. In 2016, only 19.3% of faculty members employed by schools of nursing in Canada held a permanent position.[11] This shortage is fueled, in part, by a rapidly aging workforce, an undersupply of doctorally prepared nurses to take their place, lack of qualified applicants, and poor work environment.[12–15] About 40% of Canadian nursing faculty are over the age of 55 and 17.8% are eligible to retire.[11] The retirement projections of the aging faculty limit the pool of doctorally prepared faculty as fewer nurses are applying for graduate programs.[15–18] According to Canadian Associations of Schools of Nursing,[11] the number of graduate students currently enrolled in nursing schools is far less than what is required to meet the demands for advance practice in clinical settings and in academia, and this is contributing to exponential growth in unoccupied faculty positions across Canada. This is exacerbated by the fact that only 60% of nurses with doctorates, choose the academic role.[19] Across Canada, schools of nursing identified shortage of qualified applicants, non-competitive salaries, and lack of funds to create permanent positions as the three main factors limiting their ability to recruit new faculty.[19–21] This results in heavy workloads for employed faculty, their inability to train sufficient graduate students, lack of quality time for students in the program, contributing to longer graduation periods, faculty burnout, work-life conflict (or lack of balance between work and life), dissatisfaction, and further attrition.[17,22,23]

With the concurrent nursing faculty shortage and the impact of the COVID-19 pandemic on the nursing workforce, attracting and retaining quality faculty is extremely important to educational institutions as low faculty retention rate might create both monetary and academic consequences.[24] The predominant discourse on nursing faculty recruitment and retention centers on the realities of an aging workforce, lucrative opportunities in the clinical and private sectors, heavy workloads, poor work environment, lack of organizational support,[25–27] and unrealistic role expectations.[23,28,29] What is missing in this discourse is a nuanced understanding of the joint effect of multiple work-life issues on recruitment and retention of early career researchers (ECRs)/academics, and comprehensive strategies to alleviate the shortage of faculty. Additionally, past studies on nursing faculty have mainly been in the US context with little focus in Canada.[26,30] There is limited understanding, at all administrative levels, regarding the impact of institutional work-life issues including excessive workloads, long working hours, and lack of

For peer review only - http://bmjopen.bmj.com/site/about/guidelines.xhtml

mentorship on nursing faculty members' satisfaction, and subsequent implications on their intentions to leave their job.[10]

Using Structuration theory[31] to explore the interplay between individuals and organizations, this study seeks to understand work-life (the intersection of work with personal life) experiences of nurse academics, including ECRs and the factors they identify as relevant to their success. Exploring the perspectives, concerns, and experiences of nursing faculty, especially ECRs, is important in academia in terms of succession planning. Thus, the overall goal of this study is to investigate nursing faculty's work-life experiences in Canadian academic settings and the factors that influence their retention.

Objectives

The primary objectives of this study are to: (1) assess the facilitators and barriers that influence new and early career faculty's successful transition to the academic role; (2) examine the personal and situational factors that jointly influence nursing faculty's satisfaction and intentions to remain in their jobs; (3) delineate the comparative experiences of female and male academics; (4) examine how institutional leadership impacts new faculty's quality of work-life, and productivity; and (5) explore nursing faculty's understanding of work-life integration and balance.

Theoretical perspectives

Guided by the overall goal, the proposed study is grounded in the *Structuration theory*,[31] and the relational interdependence of structure and agency (see Figure 1). The use of structuration as the theoretical lens in this research offers a multidimensional approach that takes into account the various underlying components of the "structure" which is a set of rules and resources that enable and constrain decisions, choice, action, and thought, and "agency" referring to the ability of an individual to take action, with an appreciation for their interrelatedness.[32] Structuration theory takes a dualistic view of social worlds by considering both the members of the organization (actors) and the organization's structure, and the reality of the tensions within this dialectic and the dynamic and transformational processes inherent within organizations.[31] The actions that individuals in any organization take to meet their work and non-work demands (e.g., personal/family caregiving) at a particular time are shaped by the structural properties of that context (e.g., resources, institutions).

Structuration theory is a powerful framework for studying the qualities of human relationships and a productive theoretical lens to apply to this study of work-life issues (or inter-role conflict) in academia particularly because it allows researchers to consider the different levels of influence that affect faculty's retention. Individuals' actions and interactions create or recreate the lower-level structures (e.g., at the family level), and therefore reinforce the higher-level structures at the level of organizations.[33] In other words, individuals tend to create the kinds of family structures that fit the organizational expectations as they are created. Several researchers[33–35] have measured "how" structures (e.g., leadership) can influence employees' ability to be satisfied and productive at work. The literature indicates that leadership influences the alignment of individual behaviour in an organization. Effective leadership has been shown to contribute to high morale in workplace which results in increased satisfaction and organizational commitment.[36–39] However, it is unclear how institutional leaders may bring about behavioural changes in academia. This study seeks to address this gap and the evidence from this research

For peer review only

will add to the theoretical basis for extending Giddens's structuration theory to incorporate leadership as a precursor of faculty satisfaction and retention.

The proposed study seeks to adapt and strengthen the use of this theory by exploring the role and effects of both structural/workplace and personal factors on individual's adjustment to the work environment, and the multiple dimensions of organizational and career satisfaction. By situating this study on faculty work-life within the structuration framework, evidence from this research on how the interplay of personal and situational factors culminate in either retention or attrition will have broader implications pertaining to the work-life debate among women in the Canadian workforce and beyond.

<Insert Figure 1 here>

METHODS AND ANALYSIS

Design

This study will adopt a two-phase sequential explanatory mixed-methods design — a cross-sectional survey followed by qualitative focus group interviews to answer the research objectives, given their combined strength in addressing the complexities of work-life issues in Canada.[40] The phases of the study including recruitment and data collection procedures are outlined in Figure 2.

Phase 1: Quantitative methods and analysis

The first phase of the study will focus on the quantitative survey, which will help to address ***Objectives 1, 2, 3,*** and ***4***. A comprehensive literature review on quality of work environment was conducted prior to the study which will be used to inform the survey design.

Sample

The population of interest is nursing faculty working in Canadian colleges and universities. Eligibility criteria include full-time or part-time instructional/teaching and/or research faculty (e.g., lecturer, teaching track, and tenure-track professor), including ECRs within their first five-seven years in academia. The 5-7 timeframe was chosen based on the tenure review process adopted by most Canadian universities.[41] Based on the estimated number of new and ECRs/academics in Canada,[19] a total of 1500 potentially eligible participants will be surveyed. While there is no defined formula for sample size estimation in structural equation modeling (SEM), a sample exceeding 200 subjects is recommended to maintain the accuracy of estimates, adequate power, and to ensure representativeness.[42,43] As such, a targeted sample of 200 faculty members will be a sufficient sample size to obtain reliable parameter estimates among variables in the proposed theoretical framework for this study.[44]

Recruitment and data collection

An email invitation will be sent to eligible faculty members using their publicly available e-addresses to complete a Web-based structured questionnaire offered in both Canadian national languages – English and French. The questionnaire will be hosted securely online using the author's institutional site licensed Qualtrics survey software. The email invitation will include a description about the study, study purpose and inclusion criteria and a link to the online survey.

For peer review only

The survey will contain a letter explaining the study risks and benefits as well as description of strategies undertaken to ensure confidentiality and anonymity. No personal identifiers will be collected and/or linked to individual respondents as participants will be assigned with a random PIN number to complete the survey anonymously. Participants can terminate the survey at any time as they desired prior to submitting their response without penalty. Return of a completed survey will indicate informed consent to participate. The tailored design method (TDM) for Internet surveys[45] will be used as a technique to improve survey response rates and to maximize return. The TDM involves a set of procedures intended to create respondent trust and perceptions of increased rewards with the overall goal of reducing survey error. Non-respondents will receive a reminder notice 3 weeks following the date of the initial invite, and a subsequent remainder message 4 weeks thereafter to improve participation and ensure an adequate sample size and representative sample. Other recruitment strategies and incentives will be considered including a chance for participants to enter a draw to win a prize (e.g., gift card). At the end of the survey once participants have submitted their responses, they will receive a thank you note and an option to either exist the Qualtrics platform (e.g., close the browser), or click on a separate link to be redirected to a GoogleDoc form where they will provide informed consent if they choose to partake in the qualitative phase of the study (focus group) at a later date.

Measures

Among other broad topics, the study survey will cover workload issues, mentorship, leadership practices, organizational support, and job satisfaction. Examples of standardized questionnaires to be used in this study include the well-validated Multifactor Leadership Questionnaire,[46,47] Job Satisfaction Survey,[48] and Work Interference with Personal Life (WIPL).[49–51] Sample items from the WIPL include: 'My job makes it difficult to maintain the kind of personal life I would like' and 'I often neglect my personal needs because of the demands of my work.' Issues of reliability and validity as well as survey length to minimize respondent burden will be considered in selecting the final survey items. Demographic data including age, gender, ethnicity, highest education, academic rank, and tenure status will be collected. Anticipated time to completion the survey is approximately 15-20 minutes.

Data analysis and interpretation

Descriptive statistics will be used to profile the characteristics of new nursing faculty, and to analyze bivariate relationships using SPSS (v.26) software.[52] Rigorous methods will be used to verify the accuracy and quality of the measurement tools by performing confirmatory factor analysis and Cronbach's alpha reliabilities. Given the complexity of the research questions, the target is to employ structural equation modelling (SEM) technique with maximum likelihood estimation to test the fit between the data and the hypothesized model in Mplus software (v.7.3).[44,53] SEM is a powerful multivariate technique which allows us to examine multiple relationships among variables in a single model.[54]

<Insert Figure 2 here>

Phase 2: Qualitative methods and analysis

A major critique of quantitative surveys, including the Web-based self-report survey, on work-life issues is that they do not provide in-depth accounts of the work experiences of pre-tenured faculty.[40] Therefore, in second phase of this study, qualitative descriptive research (focus group interviews) will be undertaken, to complement and enrich the quantitative findings by probing in greater detail the circumstances leading to potential turnover. This component of the study will address ***Objective 5***.

Recruitment and data collection

Focus group discussions have been characterized as a microcosm of "the thinking society", capable of revealing the processes whereby social norms are collectively shaped through debate and argument.[55,56] As such, the proposed study will make use of focus group discussion. The semi-structured focus group sessions will provide a forum for faculty members to critically discuss a range of issues including work-life balance, workload demands, supports, and resources. The sampling strategy for the focus group will consist of a purposive sample of approximately 20-25 survey respondents who volunteer to participate in the qualitative phase of the study. This is sufficient sample size for the type of design.[40] There will be five focus groups, consisting of a mix of all gender including male and female nurse academics. Each focus group discussion will have 5 members (n=25) and will be about 60 minutes in duration. The number of focus group attendees in this study is informed by the requirements of thematic/theoretical saturation, which is the point at which themes become repetitive.[57] Faculty members will be interviewed[58] virtually/online via Zoom or any other communication medium of their choice to enhance the feasibility of this phase given the national scope of the study. There will also be an option for in-person interview as face-to-face interviews offer the advantages of rapport and visual cues.[58] As a token of appreciation, focus group will receive a $25 e-gift card.

Focus group interview guide

An interview guide will be developed stemming from the Phase 1 of the study. Sample focus group questions include: 'Tell us about your first few months/years as a new professor' and 'What do you believe has had the most impact on your transition to the academic role?' After obtaining informed consent from participants, the focus group discussion will be audio recorded using Audacity® and transcribed verbatim, and the research team will perform a textual analysis of emerging themes and conceptual categories, while also looking for patterns and relationships.

Data analysis and interpretation

Initially, data analysis will focus on understanding the information, then identifying codes and categories. Thematic analysis will be conducted using open-coding techniques.[59] Codes will be applied to statements that share commonalities. Topics of discussion in response to questions, areas of agreement and disagreement, and characteristics of the discussion will be examined.[60] In addition to themes that emerge from the data, other themes (e.g., demands of teaching, and lack of clarity about tenure requirements) not included in the preliminary framework will be included in analysis inductively and the framework will be modified accordingly. The qualitative data analysis software, N-Vivo (v.12),[61] will be used extensively for data management and coding of text. Due attention will be given to rigour in this study. To maintain a high level of quality, methodological (interviews and survey) and theory triangulation will be utilized, including keeping audit trails, and engaging in bi-weekly debriefing sessions.

For peer review only

Data from the observations and interviews will be anonymized, stored securely at the institutional research data repository throughout the data life (5 years).

Patient and public involvement

Patients and/or the public were not involved in the design, or conduct, or reporting, or dissemination plans of this research.

ETHICS AND DISSEMINATION

This study has been approved by the Hamilton Integrated Research Ethics Board (HiREB) (#1477). To ensure the privacy, dignity, integrity, and anonymity of the participants, only group data will be reported. Prior to consenting to the study, participants will be provided with information about study risks and benefits and strategies undertaken to ensure confidentiality and anonymity. Participants will have the option of terminating the survey at any time as they desired prior to submitting their response.

To ensure the findings have wide accessibility and impact, the knowledge mobilization (KM) plan for this study involves diverse strategies (e.g., stakeholder engagements, publications, conferences). Target audiences for this project are policymakers and influencers (e.g., government officials, practitioners, academics, health and labour unions). KM will be ongoing throughout the project to provide these audiences with timely information about the project and create meaningful exchange of ideas among these groups. Findings from this study will be presented at academic and non-academic conferences, through open access peer-review publications, and media release to reach the widest audiences, including interested laypeople and the general public. Academic and international audiences will be reached mainly through journal publications and conference presentations and a user-friendly report will be shared with nursing associations and health agencies. Further, social media (e.g., Twitter) will be

DISCUSSION

Although there are emerging studies on nursing faculty work-life,[2,18,26] the perspectives and experiences of new and ECRs are largely missing from the current debates on the issue. This perspective is vital because early career faculty are the next generation of leaders in academia, and they can provide valuable new insights into the successes and challenges of retention in the long term. Considering the views of new faculty brings to the fore the necessity of thinking in terms of succession planning. Although the nursing faculty shortage is a multifaceted problem, a few existing studies of the crises are descriptive in nature and most studies are limited to single sites.

This study is innovative in a number of levels: First, it aims to combine quantitative and qualitative methodologies in novel ways to gain a nuanced understanding of faculty members' work-life experiences and how academic leadership influences new faculty recruitment and retention. Second, the focus on academic leadership is unique in that less is known about how leadership of nursing faculties influence new nurse academics' quality of work-life, satisfaction, and subsequent retention.[18] Third, this research aims to examine the dynamic interplay of leadership, work environment, and satisfaction by jointly focusing on: (i) individual-level characteristics including personal and psychological traits; (ii) structural characteristics including institutional support and culture, and (iii) Psychosocial attributes (e.g., coping, support) to

For peer review only

provide sophisticated and comprehensive view of the transitional experiences of new faculty and resources that will aid in their success. The existing research on nursing faculty experiences is in the US context and very little on the Canadian context. Furthermore, there is paucity of research on the ECRs' experiences and how that culminates in retention.

The proposed study will make substantive contributions to both research and practice on nursing faculty recruitment and retention, which can serve as a baseline to address the national and global nursing faculty shortage. The findings will expand knowledge on how leadership impact retention decision making in the academic context, as well as in the organizational literature. The outcomes of this study will be valuable for nursing faculty planning and decision making in the medium to long term; and will advance work-life research by using evidence-based planning to explore informed policymaking, at a broader, conceptual level and in the specific case of recruiting and retention as a proxy for local and national policies. Furthermore, findings from this study can inform policy development, implementation, and evaluation process.

While this study focuses on faculty recruitment and retention and is situated in Canada, the goal is to develop an informed policymaking framework that is applicable in the international contexts, especially countries facing similar challenges. More fundamentally, the proposed research will raise the profile of discourses on women's work-life balance, a public policy issue often overlooked at the national level. Such discussion is especially pertinent in light of the disproportionate impact of COVID-19 on women, and female academics. Specifically, this study will highlight the critical role of informed/evidence-based planning plays in society, and its importance for stabilizing and enhancing recruitment and retention of new faculty. The findings will make an original contribution to work-life studies by shedding light on important challenges, considerations, and strategies for addressing the current nursing faculty shortage in Canada. Overall, the proposed study will contribute to a gap in the literature and enhance the current evidence on nursing education and faculty well-being in Canada.

This study has certain limitations to acknowledge in relation to the design, analysis, and generalizability. First, the cross-sectional design precludes the ability to make statements of cause and effect to the evidence of co-variation in the study variables and the a-priori theoretical associations. Additionally, the use of self-reported measures has potential for response bias.[62] Common method variance may also be of concern when the same individual completes all measures, and so, multiple-items and well-designed scales will be used to decrease the likelihood of this.[62] Another potential limitation is that the data will be collected from Canadian nursing faculty, which may limit generalizability to faculty members in all disciplines and/or countries. Additionally, there are numerous other factors not included in this study that could account for nursing faculty retention. For instance, personal dispositional variables, such as resilience or coping self-efficacy, may play an important role in addition to the study variables. Lastly, the subjective nature of qualitative research has potential for recall bias thus, critical attention should be paid to rigor and trustworthiness. Further research is required to address these limitations.

Captions

Figure 1. Structuration model of the intersection of nursing academy and work-life balance (Adapted from Giddens, 1984)

Figure 2. Phases of study protocol

Contribution statement

SB conceptualized the design of the study, drafted, and revised the manuscript.

For peer review only

References

1. Fox, M. F. Women and men faculty in academic science and engineering: Social-organizational indicators and implications. *American Behavioral Scientist* **53**, (2010).
2. Candela, L., Gutierrez, A. & Keating, S. A national survey examining the professional work life of today's nursing faculty. *Nurse Education Today* **33**, (2013).
3. Reybold, L. E. & Alamia, J. J. Academic transitions in education: A developmental perspective of women faculty experiences. *Journal of Career Development* **35**, (2008).
4. Ward, K. & Wolf-Wendel, L. Academic Motherhood: Managing Complex Roles in Research Universities. *Review of Higher Education* vol. 27 (2004).
5. Howe-Walsh, L. & Turnbull, S. Barriers to women leaders in academia: tales from science and technology. *Studies in Higher Education* **41**, (2016).
6. Xu, Y. J. Gender disparity in STEM disciplines: A study of faculty attrition and turnover intentions. *Research in Higher Education* **49**, (2008).
7. Canadian Institute for Health Information [CIHI]. *Nursing in Canada, 2018: A Lens on Supply and Workforce*. (2019).
8. Simpson, R. Men in non-traditional occupations: Career entry, carrer orientation and experience of role strain. *Gender, Work and Organization* **12**, (2005).
9. Cleary, M., Horsfall, J. & Jackson, D. Mental health nursing: Transitions from practice roles to academia. *Perspectives in Psychiatric Care* vol. 47 (2011).
10. Reid, T. P., Hinderer, K. A., Jarosinski, J. M., Mister, B. J. & Seldomridge, L. A. Expert clinician to clinical teacher: Developing a faculty academy and mentoring initiative. *Nurse Education in Practice* **13**, (2013).
11. Canadian Association of Schools of Nursing [CASN]. *Registered Nurses Education in Canada Statistics*. (2016).
12. American Association of Colleges of Nursing. [AACN]. *Nurse faculty shortage*. (2014).
13. Canadian Association of Schools of Nursing. [CASN]. *Registered Nurses Education in Canada Statistics 2009-2010.* (2012).
14. Kaufman, K. Introducing the NLN/Carnegie National Survey of Nurse Educators: compensation, workload, and teaching practice. *Nursing education perspectives* **28**, (2007).
15. McDermid, F., Peters, K., Jackson, D. & Daly, J. Factors contributing to the shortage of nurse faculty: A review of the literature. *Nurse Education Today* **32**, (2012).
16. Siela, D., Twibell, K. R. & Keller, V. The shortage of nurses and nursing faculty: What critical care nurses can do. *AACN Advanced Critical Care* **19**, (2008).
17. Tourangeau, A. E., Wong, M., Saari, M. & Patterson, E. Generation-specific incentives and disincentives for nurse faculty to remain employed. *Journal of Advanced Nursing* **71**, (2015).
18. Vandyk, A., Chartrand, J., Beké, É., Burlock, L. & Baker, C. Perspectives from Academic Leaders of the Nursing Faculty Shortage in Canada. *International Journal of Nursing Education Scholarship* **14**, (2017).
19. Canadian Association of Schools of Nursing [CASN]. *Registered Nurses Education in Canada Statistics: 2012-2013.* (2014).
20. Tourangeau, A. *et al.* Work, work environments and other factors influencing nurse faculty intention to remain employed: A cross-sectional study. *Nurse Education Today* **34**, (2014).

For peer review only

21. Canadian Association of Schools of Nursing. [CASN]. *The 2018-2019 National Student and Faculty Survey*. (2020).
22. Gerolamo, A. M. & Roemer, G. F. Workload and the nurse faculty shortage: Implications for policy and research. *Nursing Outlook* **59**, (2011).
23. Yedidia, M. J., Chou, J., Brownlee, S., Flynn, L. & Tanner, C. A. Association of faculty perceptions of work–life with emotional exhaustion and intent to leave academic nursing: Report on a national survey of nurse faculty. *Journal of Nursing Education* **53**, (2015).
24. Rensselaer Polytechnic Institute. *Faculty retention proves a major challenge for universities*. (2012).
25. Beckmann, C. A., Cannella, B. L. & Wantland, D. Faculty Perception of Bullying in Schools of Nursing. *Journal of Professional Nursing* **29**, (2013).
26. Boamah, S. A., Callen, M. & Cruz, E. Nursing faculty shortage in Canada: A scoping review of contributing factors. *Nursing Outlook* (2021) doi:10.1016/j.outlook.2021.01.018.
27. Smeltzer, S. C. *et al.* Challenges to research productivity of doctoral program nursing faculty. *Nursing Outlook* **62**, (2014).
28. Gormley, D. K. & Kennerly, S. Influence of Work Role and Perceptions of Climate on Faculty Organizational Commitment. *Journal of Professional Nursing* **26**, (2010).
29. McDonald, P. J. Transitioning from clinical practice to nursing faculty: Lessons learned. *Journal of Nursing Education* vol. 49 (2010).
30. Gerolamo, A. M., Overcash, A., McGovern, J., Roemer, G. & Bakewell-Sachs, S. Who will educate our nurses? A strategy to address the nurse faculty shortage in New Jersey. *Nursing Outlook* **62**, (2014).
31. Giddens, A. The constitution of society: Outline of the theory of structuration: Elements of the theory of structuration. in *Practicing History: New Directions in Historical Writing after the Linguistic Turn* (2004).
32. Orlikowski, W. J. The Duality of Technology: Rethinking the Concept of Technology in Organizations. *Organization Science* **3**, (1992).
33. Trefalt, Š., Drnovšek, M., Svetina-Nabergoj, A. & Adlešič, R. V. Work-life experiences in rapidly changing national contexts: Structural misalignment, comparisons and choice overload as explanatory mechanisms. *European Management Journal* **31**, (2013).
34. Festing, M. & Maletzky, M. Cross-cultural leadership adjustment - A multilevel framework based on the theory of structuration. *Human Resource Management Review* **21**, (2011).
35. Place, K. R. & Vardeman-Winter, J. Where are the women? An examination of research on women and leadership in public relations. *Public Relations Review* **44**, (2018).
36. Bass, B. M. *Transformational leadership: Industrial, military, and educational impact. Transformational Leadership Industry Military and Educational Impact. Transformational Leadership Industry Military and Educational Impact* (1998).
37. Boamah, S. A. The influence of transformational leadership on nurse-reported patient safety outcomes. *Electronic Thesis and Dissertation Repository. 4401. Western University, London, ON. http://ir.lib.uwo.ca/etd/4401* (2017).
38. Cummings, G. G. *et al.* Leadership styles and outcome patterns for the nursing workforce and work environment: a systematic review. *International journal of nursing studies* **47**, 363–385 (2010).

For peer review only

39. Giddens, J. Transformational leadership: What every nursing dean should know. *Journal of Professional Nursing* **34**, (2018).
40. Sandelowski, M. Whatever happened to qualitative description? *Research in Nursing & Health* **23**, (2000).
41. Gravestock, P. & Greenleaf, E. G. *Overview of tenure and promotion policies across Canada.* (2018).
42. Kline & Rex, B. *Principles and Practice of Structural Equation Modeling, 3rd edition Guilford Press*. *The Guilford Press New York London* (2011).
43. Schumacker, R. E. & Lomax, R. G. *A Beginner's Guide to Structural Equation Modeling. Lawrence Erlbaum Associates, Inc.,Publishers* (2004).
44. Kline, R. B. *Principles and practice of structural equation modeling*. (The Guilford Press, 2011).
45. Dillman, D. A. *Mail and Internet surveys: The tailored design method--2007 Update with new Internet, visual, and mixed-mode guide*. (John Wiley & Sons, 2011).
46. Avolio, B. J. & Bass, B. M. *Multifactor Leadership Questionnaire manual*. (2014).
47. Boamah, S. A. & Tremblay, P. Examining the Factor Structure of the MLQ Transactional and Transformational Leadership Dimensions in Nursing Context. *Western Journal of Nursing Research* **41**, (2019).
48. Hackman, J. R., and Oldham, G. R. The Job Diagnostic Survey: An Instrument for the Diagnosis of Jobs and the Evaluation of Job Redesign Projects. *Report No. 4, Yale University, Department of Administration Science, New Haven, CT.* (1974).
49. Boamah, S. & Laschinger, H. Engaging new nurses: the role of psychological capital and workplace empowerment. *Journal of Research in Nursing* 1744987114527302 (2014).
50. Boamah, S. A. & Laschinger, H. The influence of areas of worklife fit and work-life interference on burnout and turnover intentions among new graduate nurses. *Journal of Nursing Management* **24**, (2016).
51. Greenhaus, J. H., Allen, T. D. & Spector, P. E. Health Consequences of Work-Family Conflict: The Dark Side of the Work-Family Interface. *Research in Occupational Stress and Well Being* vol. 5 (2006).
52. SPSS Incorporated. SPSS for Windows Standard Version 26.0. (2021).
53. Muthén, L. & Muthén, B. *Mplus user's guide (6th ed.)*. *Los Angeles* (2012).
54. Mackinnon, D. P., Lockwood, C. M. & Williams, J. Confidence Limits for the Indirect Effect: Distribution of the Product and Resampling Methods. *Multivariate behavioral research* **39**, 99–128 (2004).
55. Lunt, P. & Livingstone, S. Rethinking the Focus Group in Media and Communications Research. *Journal of Communication* **46**, (1996).
56. Morgan, D. L. *Focus Group*. (2004).
57. Strauss, A. & Corbin, J. *Basics of Qualitative Research: Techniques and Grounded Theory Procedures for Developing Grounded Theory*. *The Modern Language Journal* (1998). doi:10.2307/328955.
58. Sullivan, J. R. Skype: An Appropriate Method of Data Collection for Qualitative Interviews? *The Hilltop Review* **6**, (2012).
59. Crang, M. Qualitative methods: There is nothing outside the text? *Progress in Human Geography* **29**, (2005).

For peer review only

60. Lavis, J. N., Robertson, D., Woodside, J. M., McLeod, C. B. & Abelson, J. How can research organizations more effectively transfer research knowledge to decision makers? *Milbank Quarterly* **81**, (2003).
61. Edhlund, B. M. NVivo 10 essentials: your guide to the world's most powerful data analysis software. *Stallarholmen, Sweden: Form & Kunskap AB* (2012).
62. Spector, P. E. Method Variance in Organizational Research: Truth or Urban Legend? *Organizational Research Methods* **9**, 221–232 (2006).

For peer review only

Figure 1

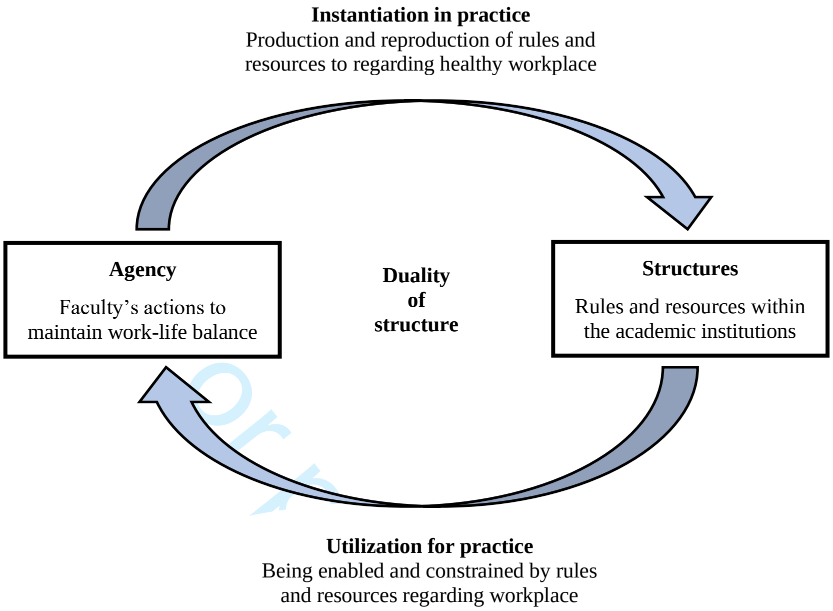

Fig. 1. Structuration model of the intersection of nursing academy and work-life balance (Adapted from Giddens, 1984)

Figure 2

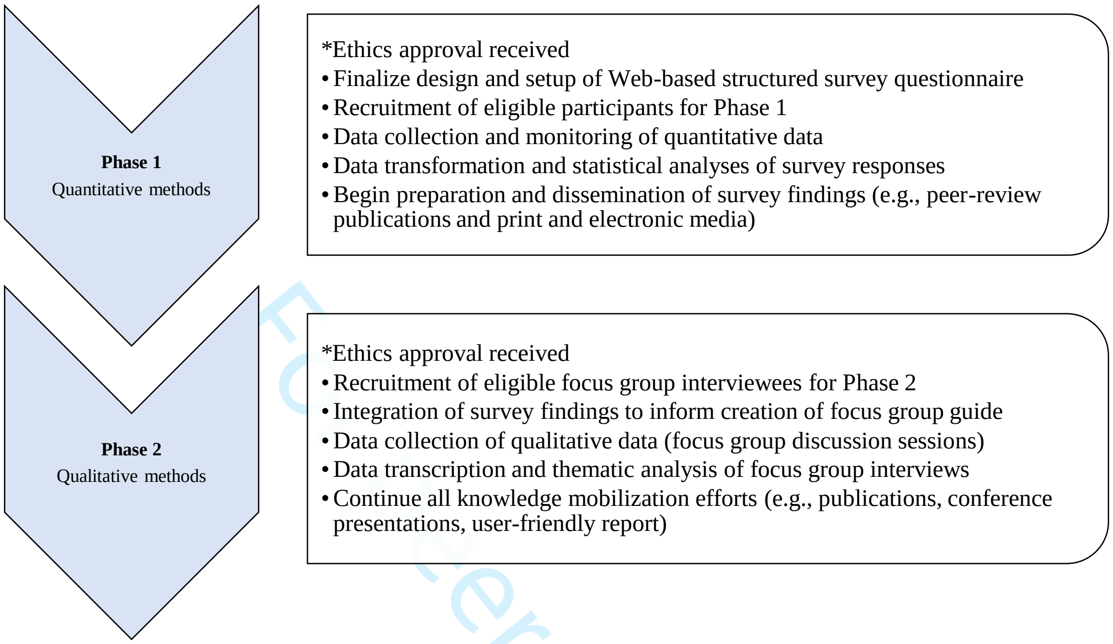

Fig. 2. Phases of study protocol

For peer review only
